# Dataset of soil hydraulic parameters in the Yellow River Basin based on *in situ* deep sampling

**DOI:** 10.1038/s41597-024-03576-7

**Published:** 2024-07-07

**Authors:** Yongping Tong, Yunqiang Wang, Jingxiong Zhou, Xiangyu Guo, Ting Wang, Yuting Xu, Hui Sun, Pingping Zhang, Zimin Li, Ronny Lauerwald

**Affiliations:** 1 State Key Laboratory of Loess and Quaternary Geology, Institute of Earth Environment, Chinese Academy of Sciences, Xi’an, 710061 China; 2 University of Chinese Academy of Sciences, Beijing, 100049 China; 3 Université Paris-Saclay, INRAE, AgroParisTech, UMR Ecosys, Palaiseau, 91120 France; 4 National Observation and Research Station of Earth Critical Zone on the Loess Plateau, 710061 Xi’an, China; 5 Department of Earth and Environmental Sciences, Xi’an Jiaotong University, Xi’an, 710049 China; 6 Xi’an Institute for Innovative Earth Environment Research, Xi’an, 710061 China

**Keywords:** Hydrology, Ecology

## Abstract

Soil hydraulic parameters are vital for precisely characterizing soil hydrological processes, which are critical indicators for regulating climate change effects on terrestrial ecosystems and governing feedbacks between water, energy, and carbon–nitrogen cycles. Although many studies have integrated comprehensive soil datasets, data quality and cost challenges result in data completeness deficiencies, especially for deep soil information. These gaps not only impede methodological endeavours but also constrain soil parameter-based ecosystem process studies spanning from local profiles to global earth system models. We established a soil dataset across the entire Yellow River Basin (YRB) (795,000 km^2^) using high-density *in situ* field sampling. This observation-based dataset contains records of soil texture (2924), bulk density (2798), saturated hydraulic conductivity (2782), and water retention curve parameters (1035) down to a maximum depth of 5 m. This dataset, which extends the recorded data range for deep soil hydraulic parameters, is valuable as a direct data resource for environmental, agronomical and hydrological studies in the YRB and regions with similar pedological and geological backgrounds around the world.

## Background & Summary

Soils serve as a crucial interface between atmosphere, biosphere, hydrosphere and lithosphere^1,2^, profoundly influencing matter and energy cycling within terrestrial ecosystems^3–5^. In particular, soil hydrological processes play a pivotal role in regulating the impact of climate change on terrestrial ecosystems and feedback mechanisms between water, energy, and carbon–nitrogen cycles^6–10^. Soil hydraulic parameters, which are in turn largely determined by soil texture and structure, serve as key factors in accurately depicting soil hydrological processes^11–13^. For instance, the saturated conductivity (Ks) is a major control of moisture movement, distribution, and fluctuations within the soil profile^14,15^. The matrix potential, which describes the strength of adhesive forces between soil moisture and solid components of the soil, determines the plant-availability of soil moisture. The soil water retention curve (SWRC), which defines the relationship between soil moisture content and matrix potential^16,17^, affects a range of processes including evaporation^18^. Thus, it is one of the fundamental attributes that characterise soil hydraulics^19^.

The main methods for acquiring the aforementioned parameters encompass *in situ* sampling^20^ and the use of pedotransfer function (PTF)^21–23^. The considerable costs of *in situ* sampling has led to a growing interest in the establishment and use of PTFs^9,24^. However, most PTFs are developed for specific regions, and their applicability to areas with different soil and climatic conditions is limited, necessitating re-calibration based on field measurements^12^. The challenges associated with obtaining soil parameters not only impede methodological endeavours, such as up-scaling based on PTFs, but also impose limitations on ecosystem process studies that rely on soil parameters^25^. These limitations affect research at a broad range of scales from site-level profile investigations^26,27^ to calibration and parametrization of comprehensive ecosystem models^2^. Therefore, the accurate measurement of soil parameters is highly beneficial for assessing soil hydrological processes not just at local scale, but also for up-scaling to regional scales, and thus facilitating multiscale ecohydrological process studies^28^.

Currently, a multitude of datasets, including Florida Soil Characterization Data^29^, WoSIS^30^, and UNSODA^31^, are dedicated to aggregating a diverse range of soil parameters derived from field measurements, comprising, in particular, the essential SWRC parameters. However, a considerable proportion of these data exhibits vague sample point coordinates and insufficient data pairs for establishing the SWRC, often lacking the wet end of the SWRC (water head ≤ 0.2 m)^24^. To address the limitations of field measurements, some studies have employed integrated PTFs to derive soil hydraulic parameters at national and global scales^32,33^. As illustrated by Gupta, Papritz^24^, integrated field measurements with PTFs to effectively globally extend the applicability of soil hydraulic parameters by supplementing missing measurement data. However, the extensive datasets mentioned above still contain limited information regarding deep soil profiles, particularly regarding the scarcity of soil information below a depth of 3 m^34^. Deep soil water, which is largely mediated by vegetation^35^, acts an important role in enabling vegetation to withstand drought stress^36,37^ and water is also a key factor affecting the soil’s ability to sequester carbon^38^. Hence, deep soil hydraulic processes play an important role in terrestrial hydrology and soil carbon budgets^39^. Given the potential impact of soil profile heterogeneity on hydraulic parameters^9,28^, which constrains the applicability of shallow soil data, it becomes necessary to broaden the depth of investigation for soil hydraulic parameters. The compilation of deep soil profile information and incorporation of detailed field records would serve as a valuable complement to existing soil datasets.

Given the limitations of the current datasets outlined above, the objective of this study was to utilise geographically precise field measurements from deep soil profiles to extend existing soil datasets with reliable deep soil property records. Furthermore, we sought to provide a quantitative foundation to facilitate the development of PTFs that rely on original data. We conducted *in situ* sampling across the entire Yellow River Basin (YRB). The YRB is extensive (795,000 km²), irrigating over 15% of China’s cultivated land and sustaining more than 12% of China’s population^40^. Furthermore, this basin encompasses most of China’s important ecological barrier belt^41^, including the Loess Plateau (LP), the world’s largest loess deposition region. Historically, severe soil erosion in this region has led to substantial loss of soil carbon to the ocean via the Yellow River, profoundly impacting the land carbon budget^42–44^. Over the past two decades, China has been one of the leading contributors to the land greening observed around the globe^45^, with the LP taking a prominent role through the “Grain for Green” program for ecological restoration^12,46^. Given the significance of the YRB for global carbon cycling, climate change, food security, and ecological stability, the investigation of soil parameters in this region does not only hold the value for regional environmental and agronomic studies, but also provide some valuable supplementation to the current global pool of soil hydraulic datasets. Moreover, our dataset offers more possibilities for ecohydrological studies including observation and modelling that focus on deep profiles by providing soil hydraulic parameters down to a profile depth of 5 m.

During three years (2008, 2018, 2019) of fieldwork, we collected a total of 2925 disturbed soil samples and 2800 undisturbed soil samples throughout the whole YRB. This extensive, and high-density observation grid contains measurements of soil hydraulic properties down to a maximum depth of 5 m. The profiles were analyzed in the laboratory, and measurements were subjected to comprehensive data quality control and cleansing processes. Furthermore, we employed the “soilhypfit” package^47^ in R (4.2.3 version) to fit the SWRC via the van Genuchten (VG) model. It should be noted that all SWRC records were derived from 10 pairs of corresponding soil matrix potential and moisture content data, covering a broad range of matrix potentials from 0.1 bar to 10 bars. For our dataset, we finally retained 2924 records of soil texture, 2798 records of soil bulk density (BD), 2782 records of Ks, and 1035 SWRC records. All records were consolidated into a unified dataset. This dataset further provides detailed meta-information for each sample, including sampling time, coordinates, elevation, depth, and land use type. We opted to preserved as much of the observed data as possible, but assigned categories of data quality which may help users to balance between quantity and quality of data depending on their research objectives and requirements. This dataset will be of value as a direct resource for environmental, agronomical and hydrological studies, as well as for calibrating PTFs. Although the spatial coverage of this dataset is limited, it covers the extensive YRB, filling the data gaps in this region and will also provide a useful data resource for studying other regions with comparable environmental setting worldwide. Finally, this dataset effectively extends the range of recorded data for deep soil hydraulic parameters around the world.

## Methods

### Study area and sampling site layout

**Fig. 1:**
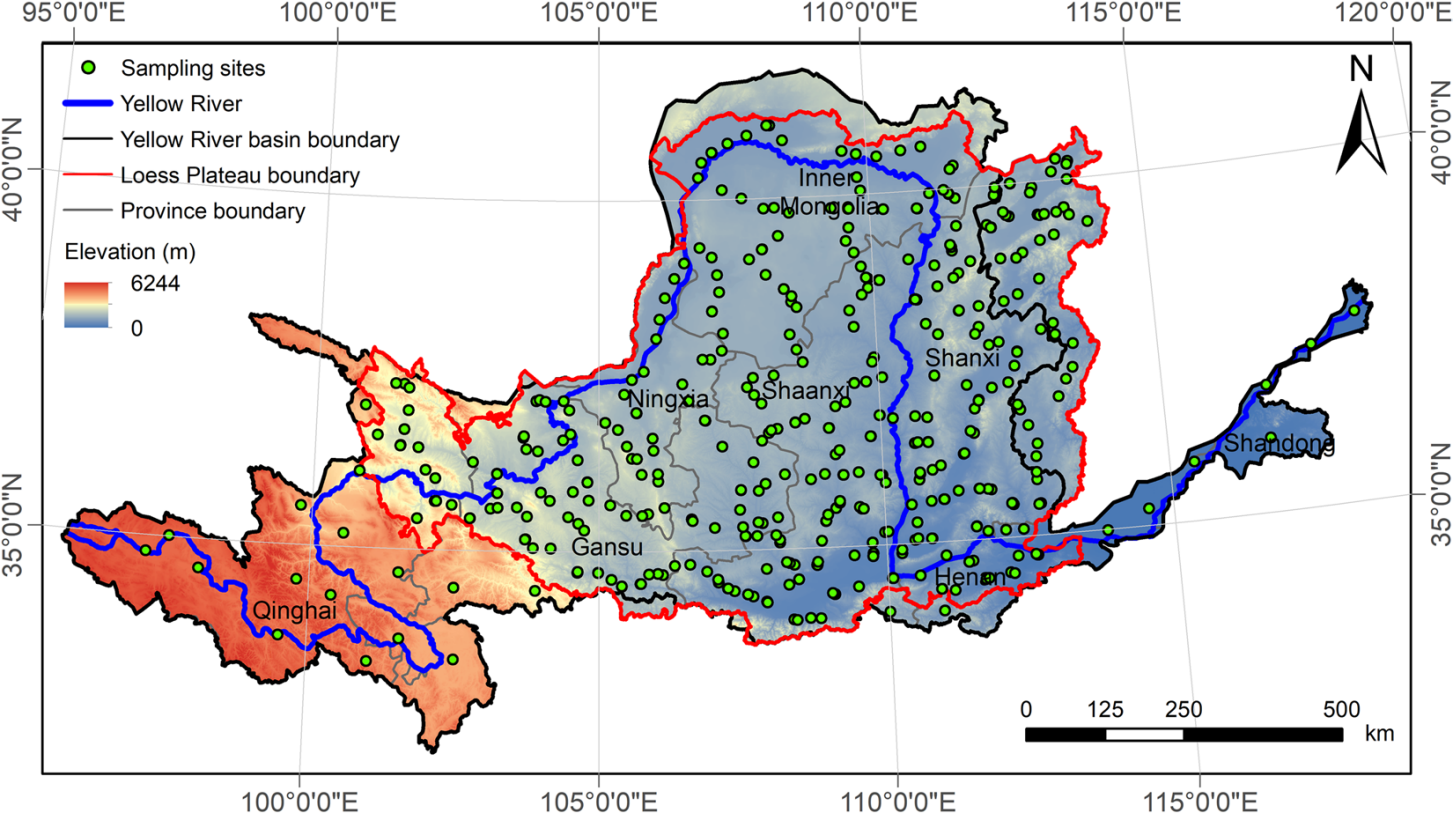
Spatial distribution of soil sampling sites in the Yellow River Basin.

The study area comprised the whole YRB (Fig. 1), which covers an approximate area of 795,000 km^2^ (95°53′–119°5′E and 32°10′–42°50′N)^2,48^. The Yellow River spans a length of 5464 km^49^, ranking as the fifth longest river in the world. We acquired disturbed and undisturbed soil samples by conducting large-scale *in situ* sampling in two phases. The first phase involved high-density shallow-profile sampling from April to November 2008. The second phase comprised medium-density deep-profile sampling conducted from September to December 2018 and from October to November 2019. We selected the sampling sites by overlaying digital maps of the sampling area by a high-density sampling grid. This grid ensured uniform partitioning of the entire basin, with the centre of each grid serving as the initial choice of the sampling site. Subsequently, the sampling locations were adjusted based on topography, soil depth, and vegetation type to increase their representativeness. Ultimately, 382 sampling sites were established in the first phase and 93 in the second phase (Fig. 1).

### Field methods

In the first phase, we excavated a 40 cm deep profile at each sampling point and collected disturbed and undisturbed soil samples from two layers (0–5 cm and 20–25 cm). During this stage, 764 disturbed and 764 undisturbed soil samples were collected. Undisturbed soil cores were placed into metal cylinders after collection to facilitate the subsequent measurement of soil hydraulic parameters^50^.

**Fig. 2:**
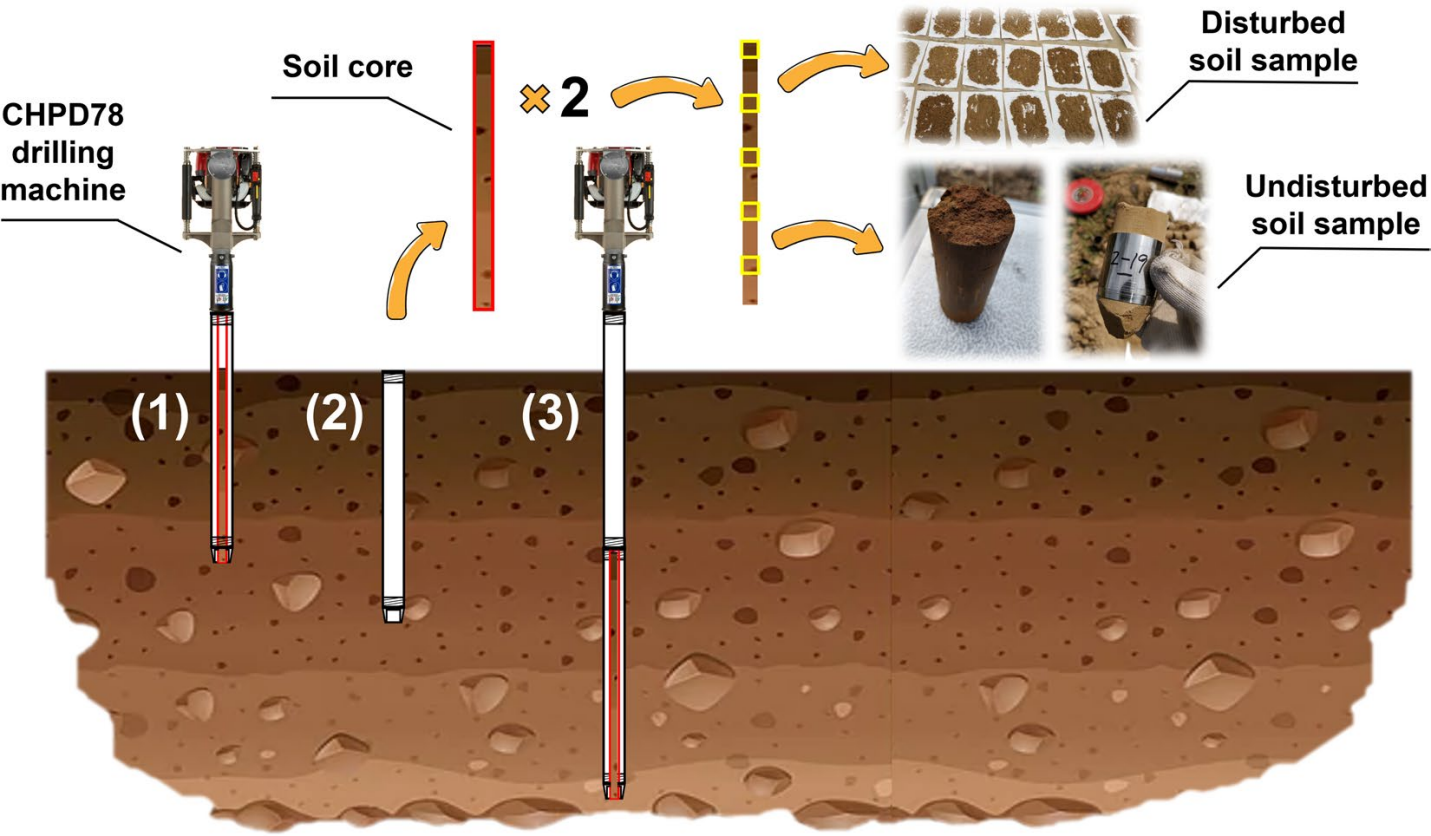
Schematic of *in situ* soil sampling using a handheld drilling machine.

In the second phase, to facilitate deep undisturbed soil sampling (the targeted depth is 5 m), we employed a hand-held drilling machine (CHPD78, Christie Engineering Pty Ltd., Australia). To prevent compression in the soil core, a dual-tube setup was used within the drilling pipe, with an inner retrievable tube designed to accommodate the soil cores. The core diameter was 37 mm, and the inner tube was replaced every 1 m during drilling to ensure the sample integrity. To ensure sample correspondence, two boreholes (with 0.5 m distance) were drilled at each sampling point to retrieve the disturbed and undisturbed soil samples (Fig. 2). For the surface layer, the disturbed and undisturbed soil samples were obtained from the depth of 0.05 m. Subsequently, the sampling was carried out every 20 cm starting from the depth of 0.2 m. During this phase, 2161 disturbed and 2036 undisturbed soil samples were collected. Owing to constraints related to soil depth and the structure in certain layers, the number of undisturbed samples was lower than that of disturbed samples. As in the first phase, the undisturbed soil samples were placed into metal cylinders after collection. To prevent samples inside the metal cylinders from disturbance, we preserved them in a shockproof foam box after sampling and promptly returned them to the laboratory for the storage. Ultimately, a total of 2925 disturbed soil samples and 2800 undisturbed soil samples were collected in the two phases.

### Laboratory methods

All disturbed soil samples underwent preprocessing, including weed removal, air-drying, grinding, and sieving (using a 1 mm mesh), before particle size distribution was analyzed. Mastersizer laser particle size analysers (Mastersizer 3000, Malvern Panalytical, UK) were used to determine soil particle size distribution. Subsequently, the soil particle sizes were categorised according to the United States Department of Agriculture (USDA) standards into clay particles (< 0.02 mm), silt particles (0.02–0.5 mm), and sand particles (> 0.5 mm), leading to the classification of soil texture following USDA standards^51^.

The undisturbed soil samples were initially immersed for 24 h to achieve full saturation. Subsequently, we performed the determination of Ks using the constant-head method^52^, which involves in maintaining a constant water head infiltration through the Mariotte bottle until a stable infiltration rate is reached. Then, the amount of water passing through the sample within a fixed time were measured to calculate the Ks. Each sample was measured three times to ensure the accuracy. Centrifuge and pressure plate instrument methods are the most widely used methods for SWRC in the laboratory^53^. The distinction between the two methods is as follows: In the low-suction range, the pressure plate method yields fewer data points and leads to a lower precision, whereas the centrifuge method provides relatively higher precision. However, the centrifuge method can be notably affected by density changes in soils with coarser textures, potentially resulting in lower precision. In the high-suction range, the pressure plate method may yield less accurate results for soils with high clay and silt contents because of inadequate drainage during the measurement process. In this case, the centrifuge method is more suitable. Considering the high silt content of most samples in this study and the time costs of pressure plate instrument method, the centrifuge method is more suitable for determining the SWRC. Utilising a centrifuge (CR21N, Hitachi, Japan), we set a series of different rotate speeds to correspond to different suction conditions (as being outlined in Table 1). After implementing each centrifugation process corresponding to different rotate speeds under a constant temperature of 20 °C, we removed the metal cylinders from the rotor, weighed, and recorded the total mass of the metal cylinders and the internal soil sample. Then, using the final measurement of the metal cylinders and dry soil mass, we calculated the gravimetric soil water contents corresponding to different suctions. Prior to measuring the SWRC, the soil saturation water content (θ_s_) was initially tested. Subsequently, the BD was assessed after oven-drying (at 105 °C for 10 h), enabling the conversion of gravimetric water content to volumetric water content.

**Table 1:** Soil matrix potential and corresponding water head range when measuring the soil water retention curve based on centrifugation.

No.	1	2	3	4	5	6	7	8	9	10
*Ψ* (bar)	0.01	0.10	0.30	0.60	0.80	1.00	3.00	6.00	8.00	10.00
*h* (mH_2_O)	0.10	1.02	3.06	6.12	8.16	10.20	30.59	61.18	81.58	101.97

### SWRC fitting and parameter acquisition based on the VG model

Upon obtaining soil water suction and volumetric moisture content data for each sampling point, we employed the “soilhypfit” package in R for fitting the SWRC using the “fit_wrc_hcc” function, in line with existing research^24^. “soilhypfit” is an R package designed for the parametric modelling of soil water retention and hydraulic conductivity data. This function allows the estimation of SWRC parameters based on the van Genuchten (VG) model^17^, with the constraint m = 1-1/n. The VG equation (Eq. 1) is as follows:

1 $$\theta (\psi )=\frac{{\theta }_{s}-{\theta }_{r}}{{[1+{(\alpha |\psi |)}^{n}]}^{m}}+{\theta }_{r}$$

where θ(ψ) (m^3^/m^3^) denotes the volumetric soil water content at matric potential ψ, and θ_s_ (m^3^/m^3^) and θ_r_ (m^3^/m^3^) represent the saturated and residual water contents, respectively. The $\alpha$ (m^−1^) is a parameter related to the inverse of air entry pressure, and n is a dimensionless shape parameter of the VG model. During the prediction process, the “fit_wrc_hcc” function estimates parameters of the SWRC from respective measurements using the maximum likelihood method, optionally subject to physical constraints on the estimated parameters, and utilises the optimisation algorithm from the NLopt library^54^ or the Stochastic Complex Evolution (SCE) algorithm^55^. According to existing research^24^, we constrained n within the range from 1.0 to 7.0 and α within the range from 0 to 100 (m^−1^) during the fitting process. Field capacity (FC) and permanent wilting point (PWP) are two key parameters that determine the soil water availability for plants and the maximum soil water-holding capacity^56^. Hence, utilising the SWRC curves derived from the fitted VG models at each point, we projected the volumetric water content corresponding to FC (−1/3 bar, −3.37 mH_2_O) and PWP (−15 bar, −152.96 mH_2_O) for these sampling locations^57^.

## Data Records

After collating the measured and predicted soil parameters, a comprehensive soil hydraulic parameter dataset for the YRB was established. This dataset encompasses sampling points spanning the entire basin in terms of horizontal spatial distribution, with 382 shallow profile points at a resolution of 40 × 40 km and 93 deep profile points at a resolution of 100 × 100 km. The dataset has been uploaded and can be accessed via the link of https://doi.pangaea.de/10.1594/PANGAEA.965004^58^.

**Fig. 3:**
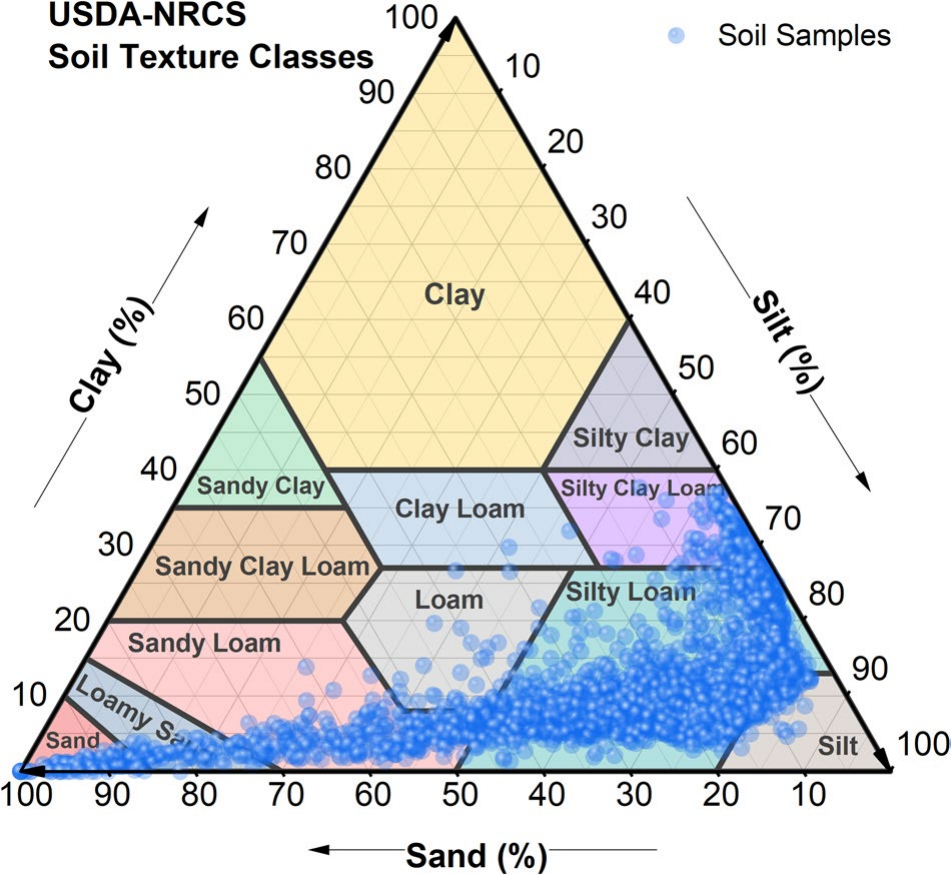
Soil texture classification of samples from the Yellow River Basin. Classification was based on USDA soil texture classification standards.

All the data in the dataset, excluding the SWRC curve parameters, were derived from direct measurements. According to USDA classification, the soil texture in the dataset falls into two major categories: loamy and sandy soils (Fig. 3).

**Fig. 4:**
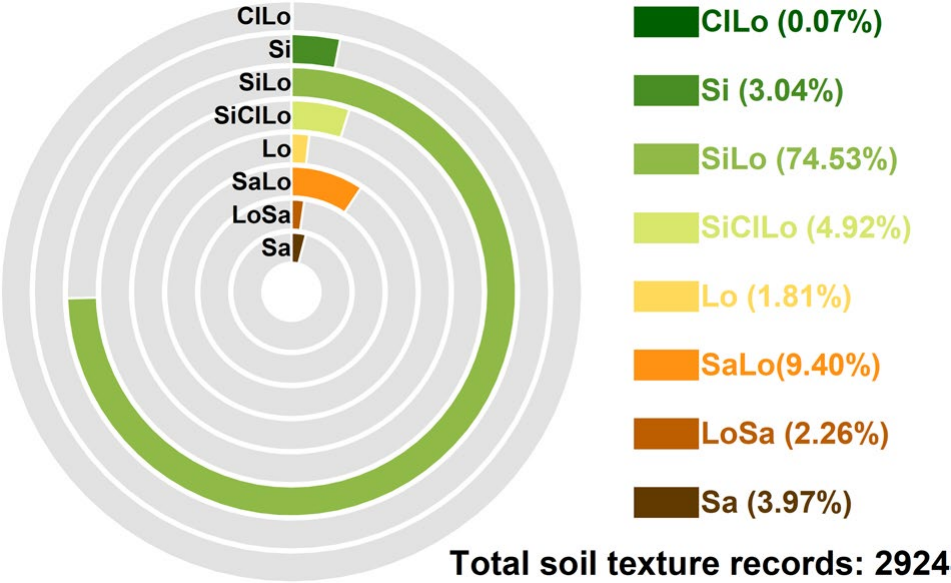
Soil texture proportion of samples from the Yellow River Basin. Sa, Sand; LoSa, Loamy Sand; SaLo, Sandy Loam; Lo, Loam; SiClLo, Silt Clay Loam; SiLo, Silt Loam; Si, Silt; and ClLo, Clay Loam.

The loamy soil category includes sandy loam, loam, silt, silty loam, silty clay loam, and clay loam. The sandy soil category includes sand and loamy sand. Among them, silty loam constituted the highest proportion (74.53%), followed by sandy loam (9.40%). The remaining soil texture classes constitute less than 5% of sampling point (Fig. 4).

**Fig. 5:**
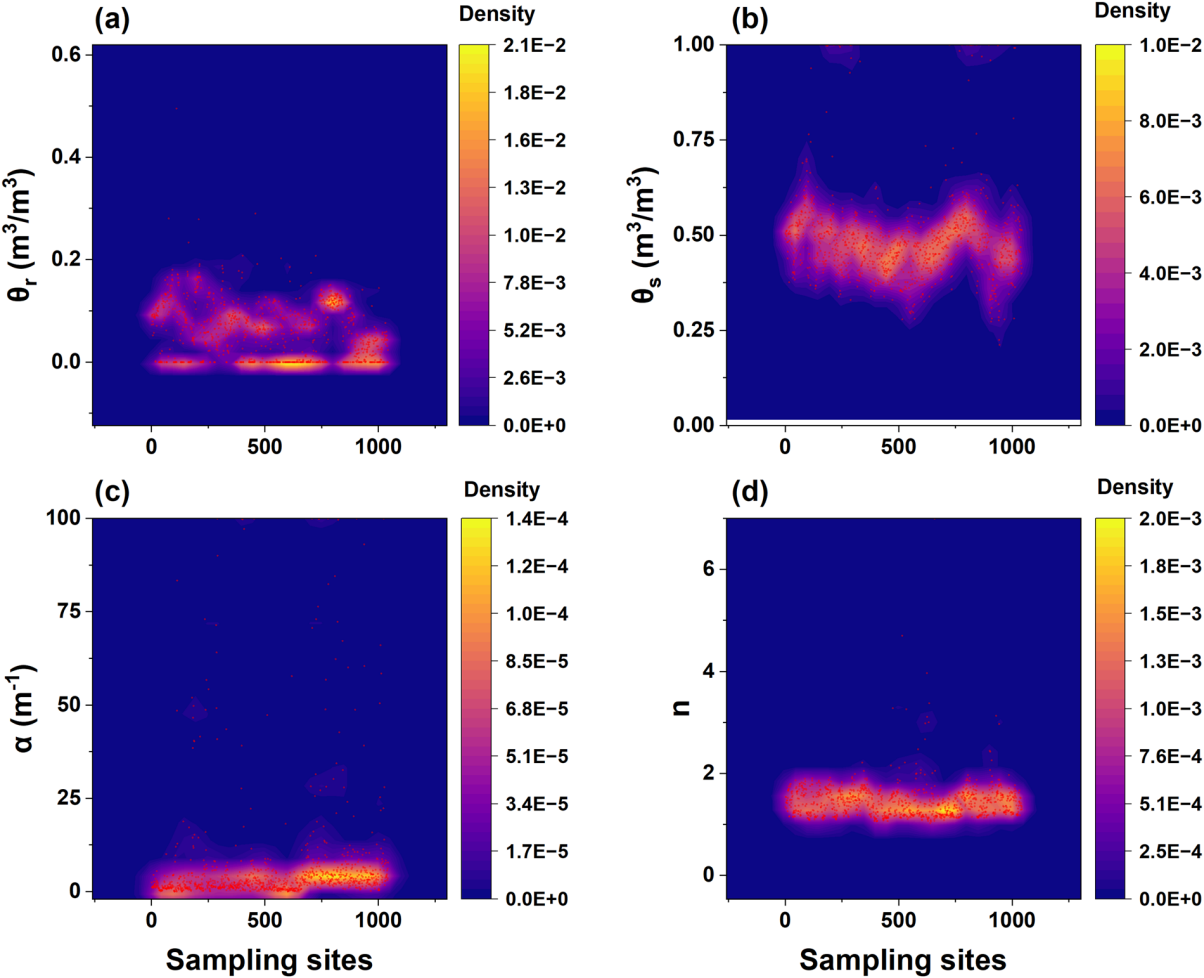
Kernel density plots of van Genuchten (VG) model parameters distribution. θ_s_ represents the saturated water content (**a**); θ_r_ represents residual water content (**b**); α (**c**) and n (**d**) are the shape parameters of the VG model.

The original data for the SWRC curve of each sampling point was also derived from direct measurements by fitting a VG model to derive the θ_s_, θ_r_, and shape parameters (α and n). According to the kernel density plot, it can be observed that the fitted VG model parameters are generally distributed within a reasonable range (Fig. 5). We applied the necessary data cleaning and quality control procedures (see Technical Validation). To preserve the integrity of the original measurement data, we introduced the relative error range information (θ_s__RE_range) into the dataset to describe the quality of the SWRC parameter fitting.

**Fig. 6:**
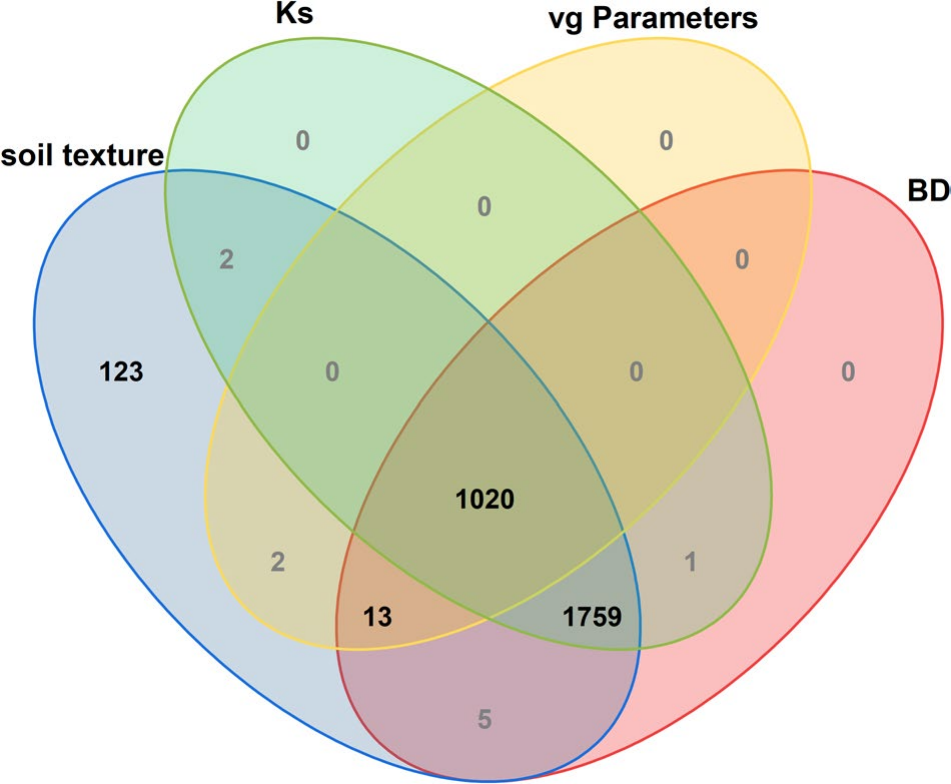
Venn diagram illustrating the number of various measurement indicators derived from soil samples in the Yellow River Basin. BD represents the soil bulk density, Ks represents the soil saturated hydraulic conductivity, and vg Parameters represents the van Genuchten model parameters.

Ultimately, 2925 disturbed field soil samples and 2800 undisturbed field soil samples were collected, and most profiles covered a depth down to 5 m. This dataset comprises 31 variables and 2925 records, and ultimately contains 2924 records for soil texture, 2798 records for soil BD, 2782 records for Ks, and 1035 records for SWRC parameters after data quality control and cleaning. A detailed description of each variable is provided in Table 2. Furthermore, a graphical representation (Venn diagram) illustrates the overlap among the different measurement indicators (Fig. 6).

**Table 2:** List of 31 variables in the Yellow River Basin soil hydraulic parameter dataset and their descriptions and units.

Header	Description	Unit
site_id	Number of the sampling site	—
sample_id	Number of samples at each sampling site	—
longitude	Longitude coordinates based on the WGS84 system	—
latitude	Latitude coordinates based on the WGS84 system	—
elevation	Elevation of the surface of the sampling site	m
land_use	Land-use type of the sampling site	—
sampling_year	The year in which the soil sample was collected	—
sampling_depth	The depth at which the soil sample was collected	m
clay	Clay (< 0.02 mm) content in soil samples	%
silt	Silt (0.02–0.5 mm) content in soil samples	%
sand	Sand (> 0.5 mm) content in soil samples	%
method_particle	Method for measuring soil particle composition	—
soil_texture_quality	Quality level of soil particle composition measuring, the levels of “A”, “B” and “C” represent high, medium and low data quality, respectively	—
soil_texture_class1	Soil texture classification based on USDA (broad categories)	—
soil_texture_class2	Soil texture classification based on USDA (subclasses)	—
BD	Bulk density of soil	g/cm^3^
method_BD	Method for measuring soil bulk density	—
Ks	Soil saturated water conductivity	cm/min
method_Ks	Method for measuring soil saturated water conductivity	—
method_SWRC	Method for measuring soil water retention curves	—
meaured_θ_s_	Measured saturated soil water content from soil samples	m^3^/m^3^
fit_θ_s_	Fitted saturated soil water content based on “soilhypfit” package in R	m^3^/m^3^
fit_θ_r_	Fitted residual soil water content based on “soilhypfit” package in R	m^3^/m^3^
fit_α	Fitted shape parameter of van Genuchten model based on “soilhypfit” package in R	m^−1^
fit_n	Fitted shape parameter of van Genuchten model based on “soilhypfit” package in R	—
fit_m	Fitted shape parameter of van Genuchten model based on “soilhypfit” package in R	—
fit_FC	Field capacity predicted by fitted SWRC curve based on “soilhypfit” package in R	m^3^/m^3^
fit_PWP	Permanent wilting point predicted by fitted SWRC curve based on “soilhypfit” package in R	m^3^/m^3^
fit_r^2^	Coefficient of determination when fitting SWRC based on “soilhypfit” package in R	—
θ_s__RE	Absolute relative error of fitted and measured saturated soil water content	%
θ_s__RE_range	Quantile range of absolute relative error of fitted and measured saturated soil water content. “Q1”, “Q3”, “Min”, and “Max” represent the “first quartile”, “third quartile”, “Q1 minus 1.5IQR”, and “Q3 plus 1.5IQR”, respectively. “-” is a symbol used to represent the range of values.	—

## Technical Validation

### Data verification and cleaning

Prior to analysis, all field-collected samples underwent a preliminary inspection to ensure the integrity and non-mixing of the disturbed samples, and the undisturbed samples in the metal cylinders were free from vibration-induced cracking or any damage. Subsequently, the original measurement data were subjected to thorough validation and data cleansing procedures. Regarding the BD, we eliminated the sample results with BD > 2.65 g/cm^3^ during the quality control process^59^. Regarding the particle size distribution data, by comprehensively referring to the existed national measurement standards and literatures^24^, we directly excluded the samples (11 records, denoted as “Error” in dataset) when the sum of particle size class contributions (clay + silt + sand) was not within 100 ± 3%. Subsequently, samples were classified based on the absolute difference between the sum of particle size class fractions and 100% as follows: Level A (< 1%), Level B (1% ≤ difference < 2%), and Level C (2% ≤ difference < 3%).

### Constraints on VG fitting

In order to assess the quality of SWRC fitting based on the VG model using the “soilhypfit” package, we computed the coefficient of determination (R^2^) for each model fit. As the “soilhypfit” package lacks a built-in function for directly calculating R^2^, we employed the following approach (Eq. 2):

2 $${R}^{2}=1-\frac{{SSE}}{{SST}}$$

where SSE represents the sum of squared errors obtained from the “ssq_wc” output of the “fit_wrc_hcc” function, while SST represents the total sum of squares total calculated based on the variances of the measured data at each point. All VG model fits yielded R^2^ values above 0.9, indicating very high fitting performance.

**Fig. 7:**
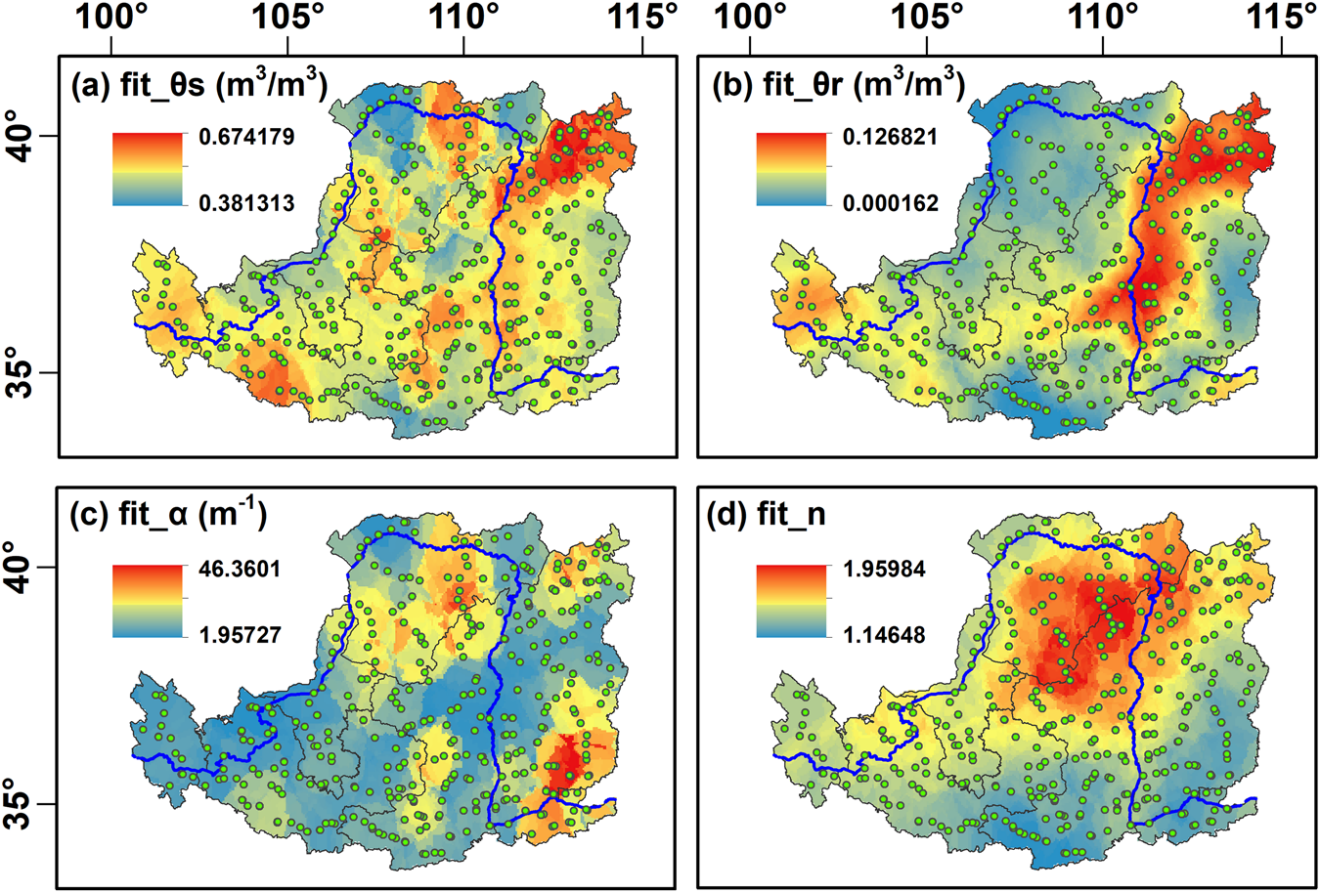
Spatial distribution of the averaged soil hydraulic parameters within 0–5 m in the Chinese Loess Plateau. θs represents the saturated water content (**a**); θr represents residual water content (**b**); α (**c**) and n (**d**) are the shape parameters of the van Genuchten model. The green dots represent our sampling sites.

To further assess the fitted data quality, we attempted to retrieve field surveys of soil hydraulic parameters from the same research area for the comparison. After filtering, we selected and plotted the spatial distribution of the mean hydraulic parameters within 0–5 m of the LP, which is the main body of the YRB (Fig. 7). The results show that the spatial distribution of θ_s_, θ_r_, $\alpha$, and n all have zonal characteristics, exhibiting obvious spatial heterogeneity. Moreover, θ_s_, α, and n have similar spatial distribution characteristics with existed investigation results^60^ in most areas within the LP, which validates the reliability of our survey to some extents.

It should be noted that finding an investigation that perfectly matches our dataset in terms of survey range, depth, and number of sample sites still presents a challenge, limiting the quantitative comparison in space. Therefore, the quality of the fitted data needs to be further assessed. Besides, the inherent limitations of the VG model for fitting to soils with high sand/clay content also need to be considered. Hence, we further calculated the relative errors between fitted θ_s_ and measured θ_s_ to quantitatively evaluate the fitting quality of each sample point. The calculation method of |RE| is as follows:

3 $$\left|{RE}\right|=\left|\frac{{\theta }_{s{fitted}}-{\theta }_{s{measured}}}{{\theta }_{s{measured}}}\times 100 \% \right|$$

**Fig. 8:**
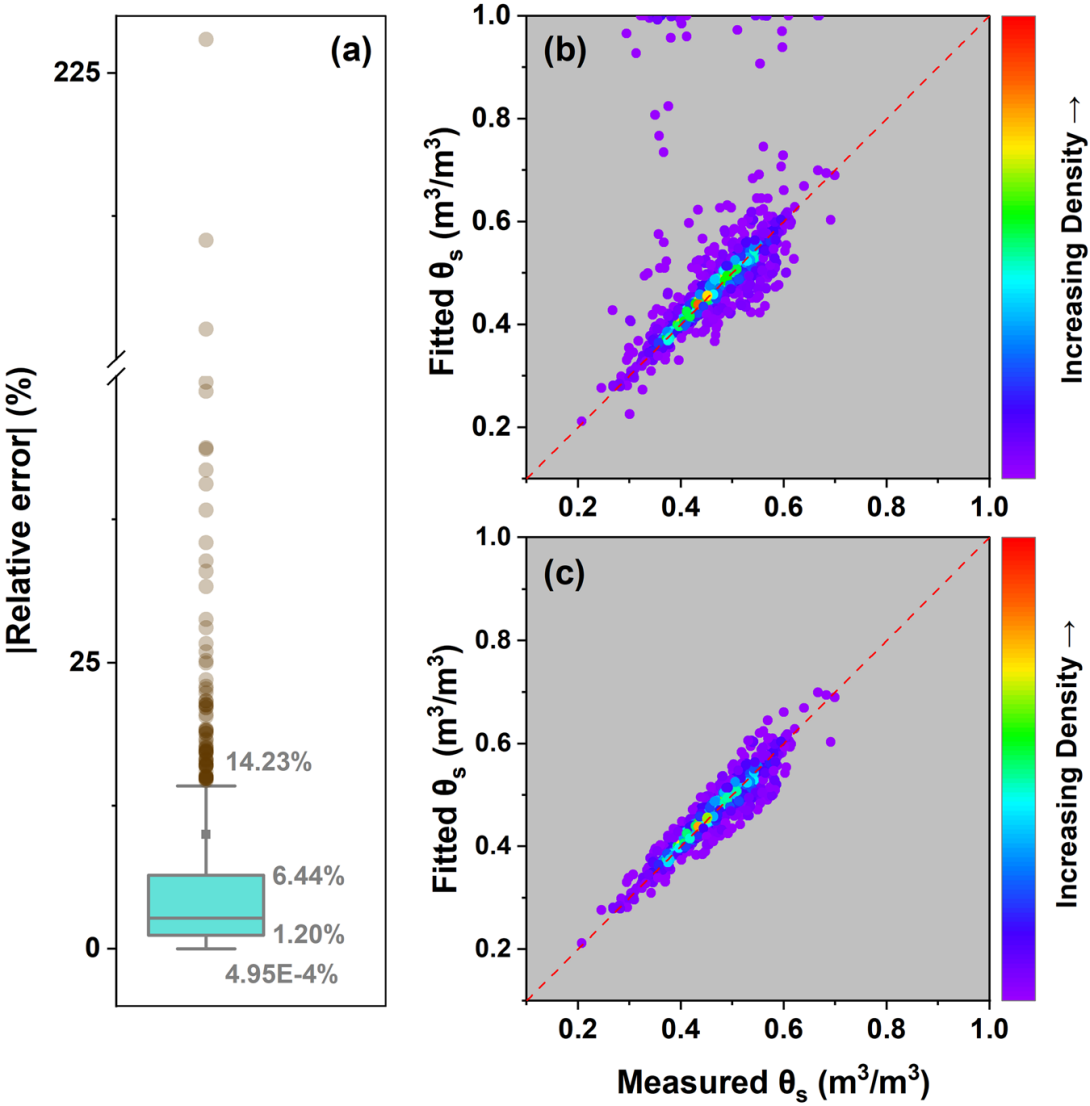
Boxplot of the absolute relative error (|RE|) between the saturated water content (θ_s_) fitted using the “soilhypfit” R package and measured θ_s_ (**a**); the distribution of measured and fitted θ_s_ for all data records (**b**); and the distribution of measured and fitted θ_s_ after removal of outliers of |RE| (**c**). The red line in the figures represents a 1:1 ratio.

Subsequently, we identified the distribution characteristics of |RE| by calculating the quartiles of these relative errors (Fig. 8a). The first (Q1) and third (Q3) quartiles were 1.20% and 6.44%, respectively. The Q3 + 1.5IQR was 14.23%, and the Q1 – 1.5IQR was 4.95E-4%. The results indicated that there were no |RE| values lower than Q1 – 1.5IQR. Upon comparing the fitted θ_s_ and measured θ_s_ before and after outlier removal, it can be observed that the points after outlier removal are largely distributed along the 1:1 line (Fig. 8b,c). Therefore, in the final dataset, we further classified the SWRC parameters according to the quartile range of |RE|, and marked the sample points where |RE| exceeds the range of Q3 + 1.5IQR as “outliers”. To retain as much of the original data as possible, we included all the RE levels in the dataset.

**Fig. 9:**
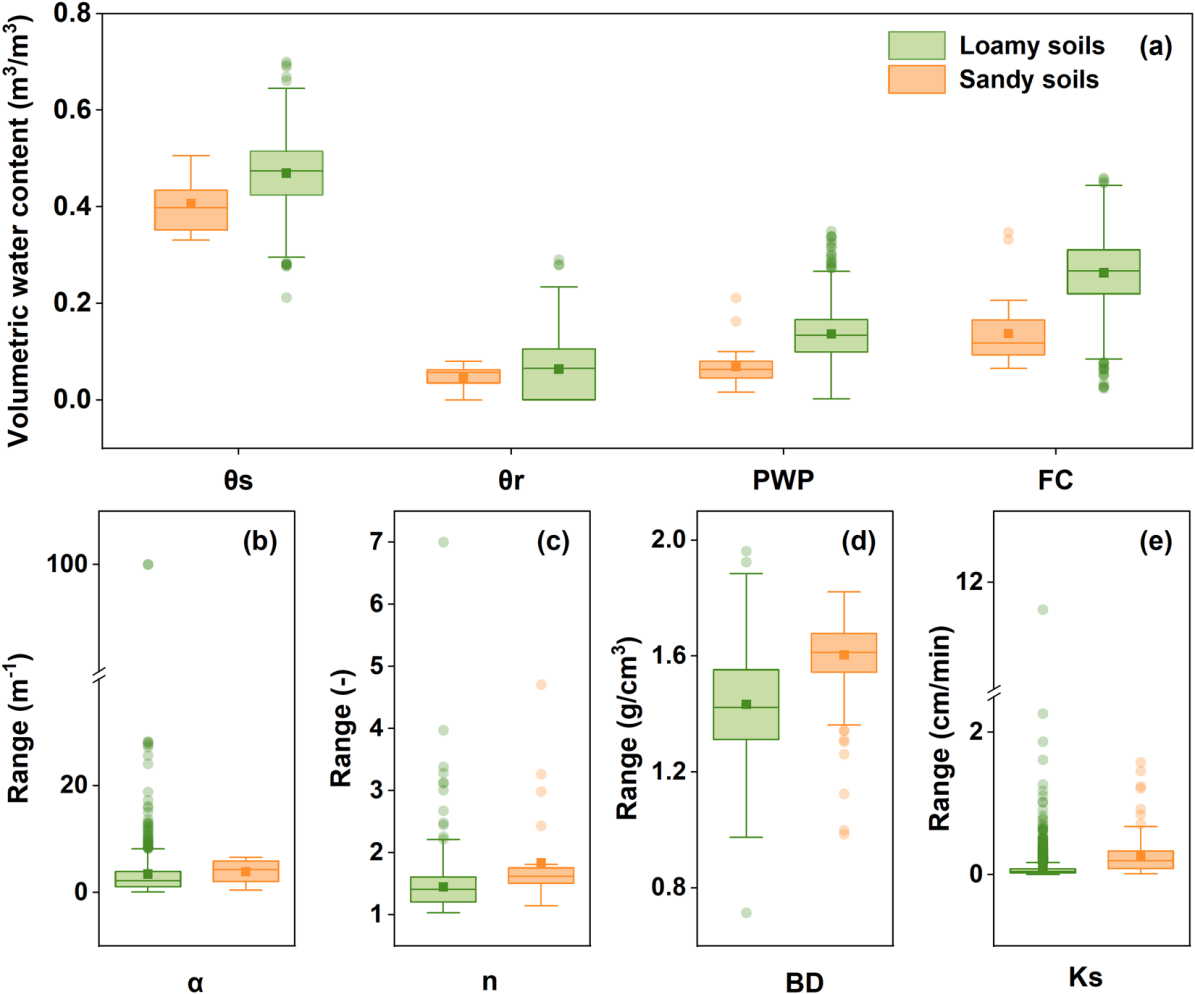
Boxplots of the distribution of hydraulic parameters for major soil texture categories. θ_s_ represents the saturated water content, θ_r_ represents the residual water content, PWP represents the permanent wilting point and FC represents the field capacity (**a**); α (**b**) and n (**c**) represent the shape parameters of the van Genuchten model; BD represents the bulk density (**d**); and Ks represents the saturated hydraulic conductivity (**e**).

By comparing the soil hydraulic parameters of the two main soil types in our datasets (loamy soil and sandy soil) after the outlier removal, we observed that for the loamy soil, all θ_s_, θ_r_, PWP, and FC were higher than those for sandy soil. In contrast, α, n, BD, and Ks for loamy soil were lower than those for sandy soil (Fig. 9). Furthermore, following the study by Goldberg *et al*.^61^, we further removed points with FC > 48% and PWP > 36%.

## Usage Notes

Considering that all data in this dataset originated from measurements of *in situ* samples, we strived to preserve the maximum number of sample test records and provided a grading system based on our quality assessment of soil texture measurements and SWRC fitting. Our intention was to allow researchers to freely choose which data to use, and choose between quantity and quality of data according to their requirements. For soil texture measurements, we suggest to use the data at Level A (< 1%) with confidence, while to use the data at level B (1% ≤ difference < 2%) and C (2% ≤ difference < 3%) selectively based on their specific requirements. For SWRC data, despite our efforts, some parameters still exceeded the predefined validity range, which included: one n parameter reached 7 and ten α parameters reached 100 m^−1^ (comprising 0.09% and 0.96% of the total valid SWRC count, respectively). Moreover, 236 θ_r_ were predicted as zero due to their inherently small actual values and 116 |RE| of θ_s_ were listed as outliers (comprising 22.8% and 11.2% of the total valid SWRC count, respectively). We recommend cautious utilisation of these records. Constrained by sampling costs, the volume of the dataset remains limited. Nonetheless, we believe that this dataset, entirely based on measured data from *in situ* samples and encompassing soil hydraulic records down to a profile depth of 5 m, can effectively address the gaps in the pool of existing observational data, and the absence of deep soil information in particular.

## Data Availability

The code used to calculate SWRC parameters can be found on Github (https://github.com/TONGYP1116/SoilHydraulicParameter.git).
